# Human Endometrial Regenerative Cells Attenuate Bleomycin-Induced Pulmonary Fibrosis in Mice

**DOI:** 10.1155/2018/3475137

**Published:** 2018-07-25

**Authors:** Yiming Zhao, Xu Lan, Yong Wang, Xiaoxi Xu, Shanzheng Lu, Xiang Li, Baoren Zhang, Ganggang Shi, Xiangying Gu, Caigan Du, Hao Wang

**Affiliations:** ^1^Department of General Surgery, Tianjin Medical University General Hospital, Tianjin, China; ^2^Tianjin General Surgery Institute, Tianjin Medical University General Hospital, Tianjin, China; ^3^Department of Ultrasonography, National Cancer Center/Cancer Hospital, Chinese Academy of Medical Sciences and Peking Union Medical College, Beijing, China; ^4^Department of Endocrinology and Metabolism, Tianjin Medical University General Hospital, Tianjin, China; ^5^Department of Anorectal Surgery, People's Hospital of Hunan Province, First Affiliated Hospital of Hunan Normal University, Changsha, Hunan, China; ^6^Department of Colorectal Surgery, The Second Hospital of Tianjin Medical University, Tianjin, China; ^7^Department of Gynecology and Obstetrics, Tianjin Medical University General Hospital, Tianjin, China; ^8^Department of Urologic Sciences, The University of British Columbia, Vancouver, BC, Canada; ^9^Immunity and Infection Research Centre, Vancouver Coastal Health Research Institute, Vancouver, BC, Canada

## Abstract

Endometrial regenerative cells (ERCs) have been recently evaluated as an attractive novel type of stem cell therapy. Previous studies have demonstrated that most ERCs accumulated in the lung after injection and are successfully used to treat diseases such as cardiac fibrosis. However, relevant studies of ERCs in idiopathic pulmonary fibrosis (IPF) have not been reported. The present study was designed to examine the effects of ERCs on bleomycin-induced pulmonary fibrosis. All IPF models in C57BL/6 mice were induced by administrating 5 mg/kg bleomycin in PBS intratracheally. ERCs were isolated from healthy female menstrual blood and were injected (1 million/mouse, i.v.) 24 hours after induction. Wet/dry weight ratio assay, hydroxyproline content, pathological and immunohistological changes, MDA content, T-SOD activity, cytokine profiles, and RT-qPCR analysis were assessed 2 weeks after disease induction. The results showed that ERC treatment significantly decreased the wet/dry ratio and reduced collagen deposition. Histological analyses, Masson staining, and hydroxyproline content analysis indicated that ERCs could reduce collagen fiber production. Immunohistochemical staining revealed lower expression of TGF-*β* after ERC treatment. Furthermore, mice treated with ERCs had lower levels of IL-1*β* and TNF-*α*, but a higher level of IL-10 in both the lung and serum. Gene expression analysis demonstrated that ERCs potently suppressed the proapoptotic gene Bax, while increasing the antiapoptotic gene Bcl-2 and antifibrosis genes HGF and MMP-9. Our results indicate that human ERCs protected the lung from pulmonary fibrosis in mice through immunosuppressive and antifibrosis effects. Moreover, these findings formed a foundation for the further use of ERCs in clinical treatment.

## 1. Introduction

Idiopathic pulmonary fibrosis (IPF), the most common form of the idiopathic interstitial pneumonias, is a chronic, progressive, irreversible, and usually lethal lung disease of unknown cause [1]. Due to no curative therapeutic options to halt disease progression and the lack of understanding of the pathogenic initiators and drivers [2], IPF becomes a most life-threatening disease with median survival time of around 2.5 to 3.5 years after diagnosis [3].

A recently held theory about the pathophysiology of IPF suggested association with oxidative stress, hyperplasia, denudation, and apoptosis of type II alveolar epithelial cells (AEC II) might be a primary cause of this process [4, 5]. Multiple alveolar injuries were regarded to be the initiator of IPF development. Injured epithelial cells release numerous mediators which not only interfere with the microenvironment of the lung but can also release a wide range of cytokines, chemokines, and growth factors, such as interleukin-1 beta (IL-1*β*), C-C motif chemokine ligand-2 (CCL2), and transforming growth factor-*β* (TGF-*β*) [6, 7]. Among those, TGF-*β* plays a central role through inducing myofibroblast accumulation, enhancing extracellular matrix synthesis, and promoting the epithelial mesenchymal transition (EMT) [8, 9]. Currently, two drugs, pirfenidone and nintedanib, were approved by the FDA for clinical use as they were able to slow down the disease progression. Among which, pirfenidone is an antifibrotic, anti-inflammatory, and antioxidant molecule which inhibits TGF-*β*-induced collagen synthesis [10, 11].

Mesenchymal stem cells (MSCs) have been recently reported as an emerging therapy for prevention of multiple lung diseases [12, 13]. Previous studies have shown that MSCs home to the sites of injury lung, inhibiting production of proinflammatory mediators, attenuating deposition of extracellular matrix, repairing epithelial tissues, and generally contributing to structural stability [14–16]. Besides that, MSCs also secreted different types of soluble factors via a paracrine mechanism to exert antiapoptosis, anti-inflammation, and antifibrosis characteristics [17, 18]. After many years of investigation, MSCs have been used in clinical treatment to treat IPF and exerted relatively curative effect [19]. However, there are still some restrictions such as the invasive acquire access, promoting tumor growth [20] and increasing the metastatic potency of cancer cells [21] in the process of MSC treatment.

Endometrial regenerative cells (ERCs), a novel type of adult stem cells derived from human menstrual blood, were first reported by Meng et al. in 2007 [22]. This new source of stem cell possesses the following advantages over conventional stem cells such as having plenty of sources, being easy access to obtain, having strong proliferation capacity, having the ability to differentiate into three germ layers in specific induction media, being able to promote tissue repair, and having the capacity to reduce the occurrence of immune rejection as a result of autologous cells transplantation [23–25]. We and others have founded that ERCs were effective in preventing critical limb ischemia [23], prolonging of the cardiac allograft survival [24], reducing renal ischemia reperfusion injury [25], and attenuating experimental colitis [26], burn injury [27], acute liver injury [28], and acute lung injury [29]. It is worth mentioning that ERCs have been investigated for the treatment of acute respiratory distress syndrome (ARDS) and located in lung tissue by the action of chemokines [29].

However, whether ERCs could be feasible for antiapoptosis, anti-inflammation, and antifibrosis in IPF remains obscure. The present study was designed to explore the effect of human ERCs on attenuating bleomycin-induced pulmonary fibrosis in mice.

## 2. Materials and Methods

### 2.1. ERC Collection, Isolation, and Culture

The isolation of ERCs was prepared according to the methods described previously [30, 31]. Briefly, after approval by the ethics committee and obtaining informed consent, ERCs were isolated from menstrual blood of healthy female volunteer donors (20–40 years old) on the first day of menstruation using a menstrual cup. Mononuclear cells from menstrual blood were separated by using a standard Ficoll method and then suspended in Dulbecco's modified Eagle's medium (DMEM) high glucose supplemented with 1% double antibiotics (penicillin/streptomycin) and 10% fetal bovine serum (FBS). The resultant cells were seeded in 10 cm dishes cultured in a humidified incubator at 37°C in 5% CO_2_. Nonadherent cells were removed by rinsing cells with culture medium, and ERCs were adhered to plastic dishes showing spindle-shaped morphology. When ERCs reached 80% confluence, they were routinely passaged using 0.25% trypsin and fourth-passage cells were used for the experiments.

### 2.2. Animals

Naive female C57BL/6 mice (Aoyide Co., Tianjin, China) weighing 18–20 g and aged 6–8 weeks were used. Animals were maintained under constant temperature and humidity conditions with a 12 : 12 light-dark cycle in the animal care facility of Tianjin General Surgery Institute (Tianjin, China) and had free access to standard chow and drinking water. All the experiments with animals were conducted in accordance with the protocols approved by the Animal Care and Use Committee of Tianjin Medical University (Tianjin, China) according to the Chinese Council on Animal Care guidelines.

### 2.3. Experimental Groups

IPF in mice was induced as previously described [32, 33]. In brief, 18 mice were randomly divided into three groups (*n* = 6) as follows:(1) sham control group: after intraperitoneal anesthesia with 10% lidocaine, mice were operated by opening and closing the skin and tissue in front of the trachea without intratracheal injection; (2) untreated group: each C57BL/6 mouse was intratracheally administered 5 mg/kg bleomycin sulfate (Beijing Solarbio Science & Technology Co. Ltd., Beijing, China) dissolved in sterile PBS in 50 *μ*l volume for induction of pulmonary fibrosis. 24 hours later, 200 *μ*l PBS was injected via the tail vein; and (3) ERC-treated group: IPF in mice was induced in the same manner as ahead. One million ERCs suspended in 200 *μ*l of PBS were injected via the tail vein 24 hours after disease induction. All mice were sacrificed on day 14 after BLM induction, and blood was obtained via the abdominal aorta. The lung was then removed for analysis or stored frozen at −80°C.

### 2.4. Wet/Dry Weight Ratio Assay

The wet/dry (W/D) measurement was used to detect pulmonary edema. After a thoracotomy, the right inferior lobar lungs were collected and weighed immediately to determine the wet weight. Then, putting the lung tissues into the incubator at 60°C for 24 hours, the dry weight was recorded.

### 2.5. Collagen Content Assessment by Hydroxyproline Assay

Hydroxyproline (HYP) assay was used to measure the collagen content in the lung homogenates using a commercially available kit (Nanjing JianCheng Bioengineering Institute, Nanjing, China). The experimental procedure was performed in accordance with the protocols provided, and the absorbance of each sample was read at 550 nm by a microplate reader. Assuming that collagen contains an average of 13.5% hydroxyproline [34], the data were expressed to quantitatively analyze the collagen content in lung tissue.

### 2.6. Histological Examination and Masson's Trichrome Staining

The left lungs were fixed with 10% formalin for 48 h and underwent the process of dehydration, paraffin embedding, and sectioning. The 5 *μ*m thickness sections were stained with hematoxylin and eosin (HE) and Masson's trichrome stain for collagen deposition according to standard protocols. According to classical Masson staining, collagen fibers are stained blue and nuclei are dyed black, while cytoplasm and muscle are dyed red.

The severity of lung fibrosis was assessed by measuring the Ashcroft score [35] for semiquantitative analysis of histological fibrotic changes. Each section was observed in 5 fields of view, and the average score of fibrosis ranging from 0 (normal lung) to 8 (total fibrous obliteration of the field) was used to evaluate [36].

### 2.7. Immunohistochemistry

To evaluate engraftment of TGF-*β*1, the paraffin specimens were cut into 5 *μ*m, following by dewaxing and rehydration. Tissue sections were incubated with 3% H_2_O_2_ to block endogenous peroxides, and antigen retrieval was processed by heating in a microwave. After enclosed by 5% bovine serum albumin (BSA), the specimens were stained with rabbit anti-mouse TGF-*β*1 polyclonal antibody (Abcam, Shanghai, China) and goat anti-rabbit IgG polyclonal antibody sequentially. And then horseradish peroxidase-conjugated avidin and brown-colored diaminobenzidine were used to visualize the labeling. Finally, the slides were restained with hematoxylin and mounted.

### 2.8. Measurement of Total Superoxide Dismutase (T-SOD) Activity and Malondialdehyde (MDA) Content

Partial lung tissues of each group frozen in −80° were collected and homogenized and then centrifuged for 10 minutes at 3000 rpm at 4°. The supernatant was extracted and tested for T-SOD activity and MDA content. The procedures used to quantify T-SOD activity and MDA content were conducted according to the manufacturer's instructions (Nanjing Jiancheng Bioengineering Institute, Nanjing, China). The absorbance changes at 550 and 532 nm were monitored.

### 2.9. ELISA

Supernatants of homogenized lung tissues and serum were used to measure the levels of IL-1*β*, TNF-*α*, and IL-10. Conducted by the ELISA kit protocol (eBiosciences, San Diego, CA, USA), each sample was added into two wells to eliminate the error, followed by methods of double-antibody sandwich, biotin amplification system, and horseradish peroxidase catalytic substrate. The reaction was terminated by addition of acid and absorbance was measured at 450 nm. The concentration of each sample was obtained by comparing the sample and the standard curve.

### 2.10. RT-qPCR Analysis

Total RNA was extracted from lung tissue using an animal tissue RNA extraction kit (DP431, Tiangen Biotech Co. Ltd.). The extracted RNAs were reverse transcribed into cDNAs by using a FastQuant RT kit with gDNase (KR106, Tiangen Biotech Co. Ltd.). The purity and quality of the RNA were examined with an UV spectrophotometer at 260 and 280 nm. To assess the expression of target genes, quantitative real-time PCR was performed using SuperReal Color PreMix (FP216, Tiangen Biotech Co. Ltd.) according to the manufacturer's instruction. Sequences of the mouse gene-specific primers used were listed as follows: *β*1-actin: forward, AGACAGGGGCCTTTTTGCTAC, reverse, AATTCGCCGGAGACACTCG; Bax: forward, AACAGGGGCTTTACGTTCACT, reverse, CGTCCCTTTATAGCTGCCTCC; Bcl-2: forward, AGCCCTGTGCCACCATGTGTC, reverse, GGCAGGTTTGTCGACCTCACT; HGF: forward, AACAGGGGCTTTACGTTCACT, reverse, CGTCCCTTTATAGCTGCCTCC; MMP-9: forward, GGACCCGAAGCGGACATTG, reverse, CGTCGTCGAAATGGGCATCT. The relative expression of the target gene was analyzed using the comparative 2^−∆∆CT^ method.

By subtracting the Ct value of *β*-actin from that of the target gene, we obtained △Ct, whereas △△Ct was calculated by subtracting the value of △Ct from that of the sham control sample.

### 2.11. Statistical Analysis

Statistical analysis was performed in SPSS 22.0. All data were presented as mean ± standard error of the mean (SEM). The data obtained from the sham control group, untreated group, and ERC-treated group were compared using one-way analysis of variance (ANOVA), following by the independent-samples *t* test to analyze parametric data. Differences with *p* values of less than 0.05 were considered statistically significant.

## 3. Results

### 3.1. ERCs Ameliorate Alveolitis and Abrogate Collagen Deposition in Lungs

**Figure 1:**
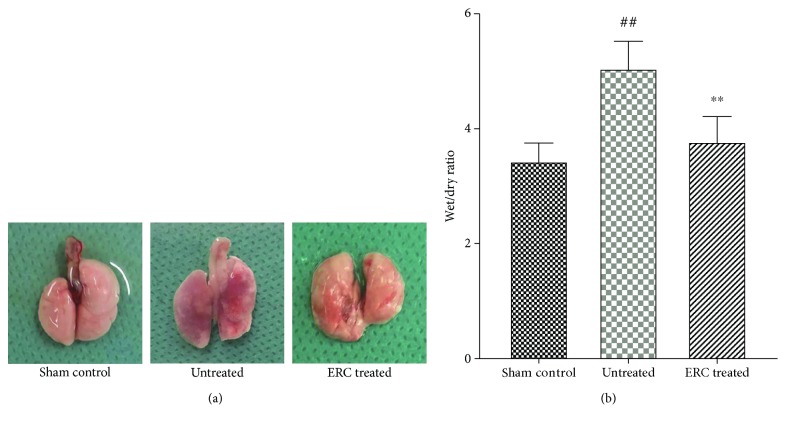
ERCs ameliorate alveolitis and abrogate collagen deposition. (a) Representative figure of gross pathology. The lung in the sham control group presents as complete and smooth, while in the untreated group became inflamed and mauve and had fibrous deposition. After ERC treatment, it showed the reverse of the inflammation and partial fibrotic patches. (b) Wet/dry ratio evaluates the pulmonary edema which represents the severity of inflammation. ERCs reduced the level of wet/dry ratio in comparison with that of the untreated group. Bar graphs represent mean ± SEM of three separate experiments. *p* values were determined by one-way ANOVA. Data shown are representative of three separate experiments performed. (^##^*p* < 0.01 versus the sham control group. ^∗∗^*p* < 0.01 versus the untreated group, *n* = 6). ERCs: endometrial regenerative cells.

It has been observed that on-going AEC injury in the lungs and aberrant repair mechanisms have been implicated in the pathogenesis of IPF. We have investigated whether human ERCs (which exhibit immunomodulatory properties) have therapeutic effects on inhibiting pulmonary fibrosis in a mouse model and found that the lung in the untreated group became inflamed and mauve and had fibrous deposition under macrography (Figure 1(a)), suggesting that bleomycin had induced severe pulmonary fibrosis. Whereas, the lung structural changes in the ERC-treated group possessed a significant relief by contrast, which were not significantly different from those in the sham control group (Figure 1(a)). In addition, wet/dry weight ratios were calculated to evaluate the pulmonary edema caused by the inflammatory responses. As anticipated, there was no significant difference in the ratios between the ERC group and sham control group. In contrast, the ERC group showed notably less pulmonary edema than the untreated group (Figure 1(b), *p* < 0.01). The results suggested that transplantation of ERCs could attenuate the levels of the inflammation by reducing pulmonary edema.

### 3.2. ERCs Modulate Cell Infiltration, Inflammatory Response, and Extracellular Matrix Remodeling in the Lungs

**Figure 2:**
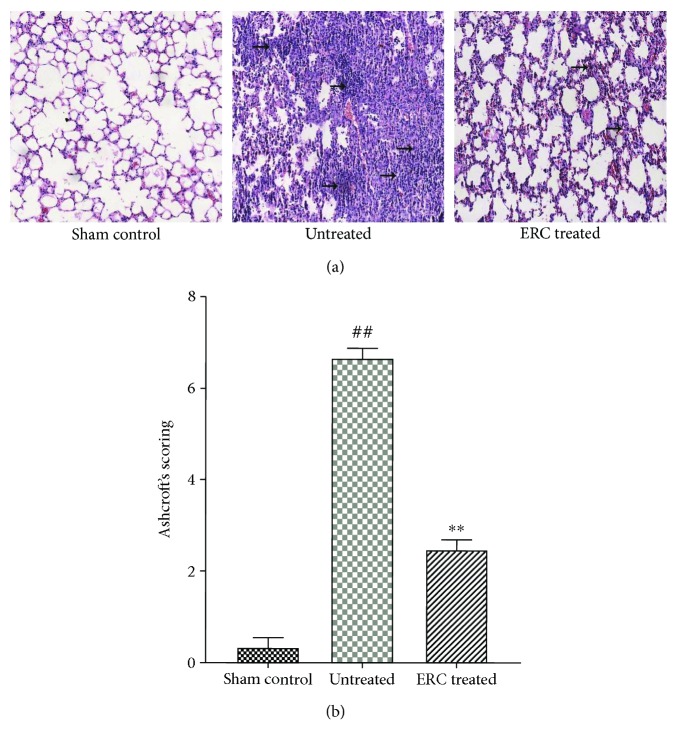
ERCs significantly attenuate pathological damage induced by BLM in mice. (a) Representative section of different groups of the lungs (200x magnification, HE staining). The lungs in the untreated group exhibited more cell proliferation, inflammatory response, and extracellular matrix remodeling than those in the normal control groups, whereas, there was a marked remission in the ERC-treated group. (b) Quantitative assessment of lung damage was performed according to Ashcroft's scores. Values represent mean ± SEM. (^##^*p* < 0.01 versus the sham control group. ^∗∗^*p* < 0.01 versus the untreated group, *n* = 6). ERCs: endometrial regenerative cells; BLM: bleomycin.

To more specifically study the lung injury, we examined detected histopathological changes of the lungs in different groups. As shown in Figure 2(a), the alveolar structure in the sham control group was clear and complete. Whereas, in the untreated group, the normal alveolar structure disappeared, characterized by the presence of extensive mesenchyme expanded with increased numbers of inflammatory cells, and there even exhibited a majority of consolidation in the lung (Figure 2(a)). Compared with the untreated group, after ERC treatment, the inflammatory cell infiltration and the interstitial fibrosis formation were markedly reduced. Even though there were still little interstitial hyperplasia, the alveolar structure remained complete. In addition, Ashcroft's scores were performed in a blinded fashion to quantify lung damage (Figure 2(b), *p* < 0.01, the untreated group versus the ERC-treated group), which correlate with the pathological changes of the lung tissues.

### 3.3. Localization and Quantitative Detection of Fibrous Tissue Changes in the Lung

**Figure 3:**
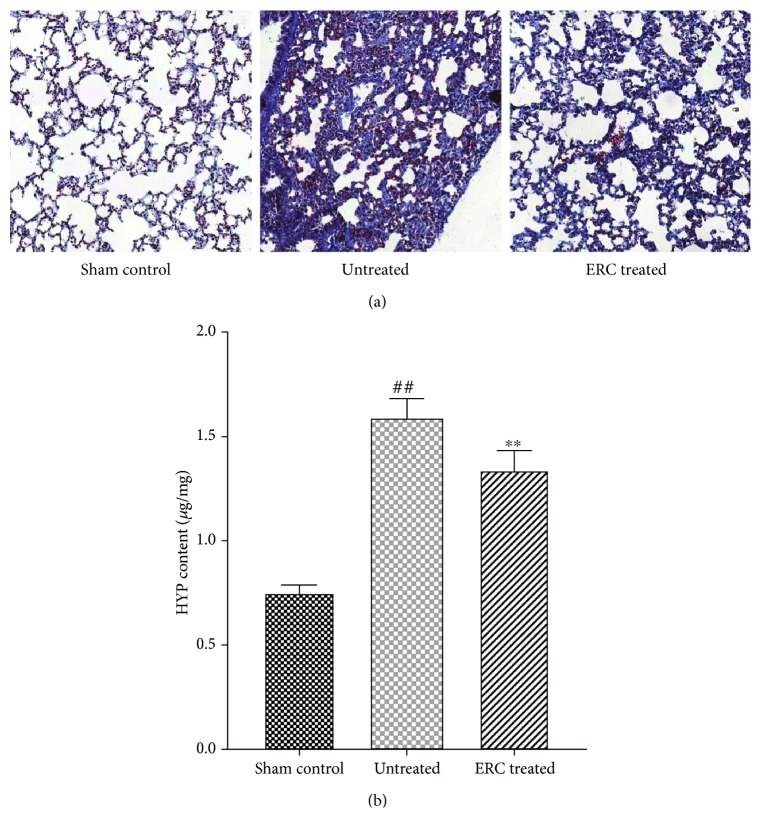
Focus on fibrous tissue changes in the lung. Representative images of Masson staining and hydroxyproline assessment. (a) The ERC-treated group exhibits smaller collagen fiber production, interstitial hyperplasia, and alveolar structural damage than the untreated group (200x, Masson staining). (b) Levels of collagen deposition in the lung are determined by hydroxyproline estimation. On treatment with ERCs, a reduction in levels of hydroxyproline was observed. Values represent mean ± SEM. (^##^*p* < 0.01 versus the sham control group. ^∗∗^*p* < 0.01 versus the untreated group, *n* = 6). ERCs: endometrial regenerative cells.

To investigate the location and content of fibrous tissue changes in the lung, we performed Masson staining on each group. As shown in Figure 3(a), a few collagenous fibers (blue staining) were presented in the sham control group without the presence of interstitial fibroplasia. With regard to the untreated group, a large quantity of collagenous fibers was observed (Figure 3(a)), whereas, after ERC treatment, the collagen fiber production reduced. Meanwhile, the interstitial hyperplasia and alveolar structural damage were significantly decreased in the lungs. To further verify our findings, we determined the level of lung hydroxyproline, a major component of collagen which contains an average of 13.5%. In the untreated group, hydroxyproline content (Figure 3(b)) was significantly higher than that in the sham control group (*p* < 0.01). However, the content was decreased after ERC treatment (Figure 3(b), *p* < 0.01, the untreated group versus the ERC-treated group). These findings suggested that ERCs could reduce production of fibers and promote recovery of fibrosis in the lung.

### 3.4. ERCs Reduce the Expression of Initiation Factor TGF-*β* in Promoting Fibrosis

**Figure 4:**
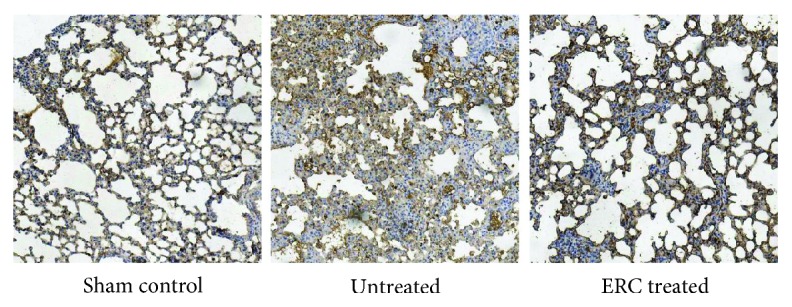
ERCs attenuate lung fibrosis by reducing the expression of vital factor TGF-*β*. Immunohistochemical result of transforming growth factor-beta (TGF-*β*) (×200 magnification); yellowish-brown staining signifies positive staining of TGF-*β*. In the untreated group, TGF-*β* expressed abundantly and mainly deposited on proliferating inflammatory cells and fibrous tissues, while in the ERC-treated group, it can be seen that the TGF-*β* expression is reduced, which is similar to the sham control group. ERCs: endometrial regenerative cells; TGF-*β*: transforming growth factor-*β*.

Subsequently, we attempted to ascertain the ability of ERCs to inhibit lung fibrosis and to study whether its underlying mechanism was associated with the vital factor TGF-*β*, which functions as a central regulator in promoting fibrosis. We intuitively tested its expression level in lung tissue by immunohistochemistry. As shown in Figure 4, high density of TGF-*β* staining was detected in the untreated group. In sharp contrast, remarkably weaker TGF-*β* staining was found in lung tissue of the ERC-treated group, which was indistinguishable from that of the sham control group. This finding demonstrated that treatment with ERCs might alleviate pulmonary fibrosis by decreasing the secretion of TGF-*β*.

### 3.5. ERC Treatment Improves the Antioxidant Capacity of the Lung Tissue

**Figure 5:**
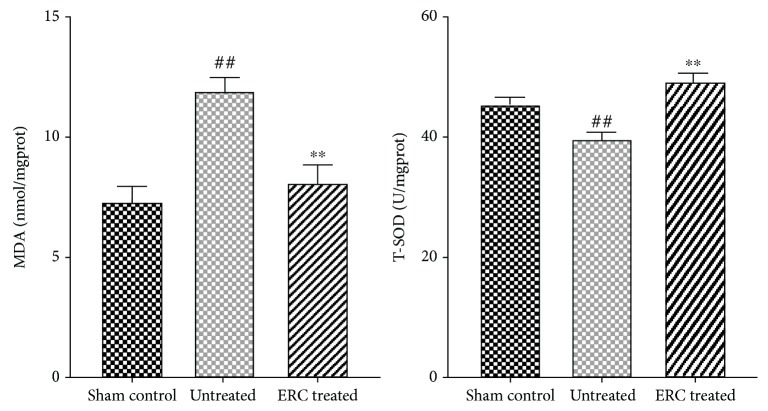
ERC treatment improves the antioxidant capacity of the tissue. Pulmonary MDA and T-SOD activity were detected by a colorimetric method. Data revealed that ERC treatment improves T-SOD activity, whereas it reduced the content of MDA in lung homogenate. Values represent mean ± SEM. (^##^*p* < 0.01 versus the sham control group. ^∗∗^*p* < 0.01 versus the untreated group, *n* = 6). ERCs: endometrial regenerative cells; MDA: malondialdehyde; T-SOD: total superoxide dismutase.

Total superoxide dismutase (T-SOD) plays an important role in the oxidation and antioxidant balance of the organism. The enzyme can scavenge superoxide radicals and protect cells from injury. The amount of malondialdehyde (MDA) reflects the extent of lipid peroxidation in the body and indirectly reflects the extent of cell damage. As shown in the result, T-SOD activity in the lung tissue from the ERC-treated group was significantly increased compared to that from the untreated group (*p* < 0.01, Figure 5), whereas, the amount of MDA in ERC-treated group was markedly lower than that in the untreated group (*p* < 0.01, Figure 5). Thus, the data suggested that treatment with ERCs might attenuate the oxidative stress in pulmonary fibrosis.

### 3.6. ERC Treatment Delays the Fibrosis Progress by Regulating Cytokine Expression

**Figure 6:**
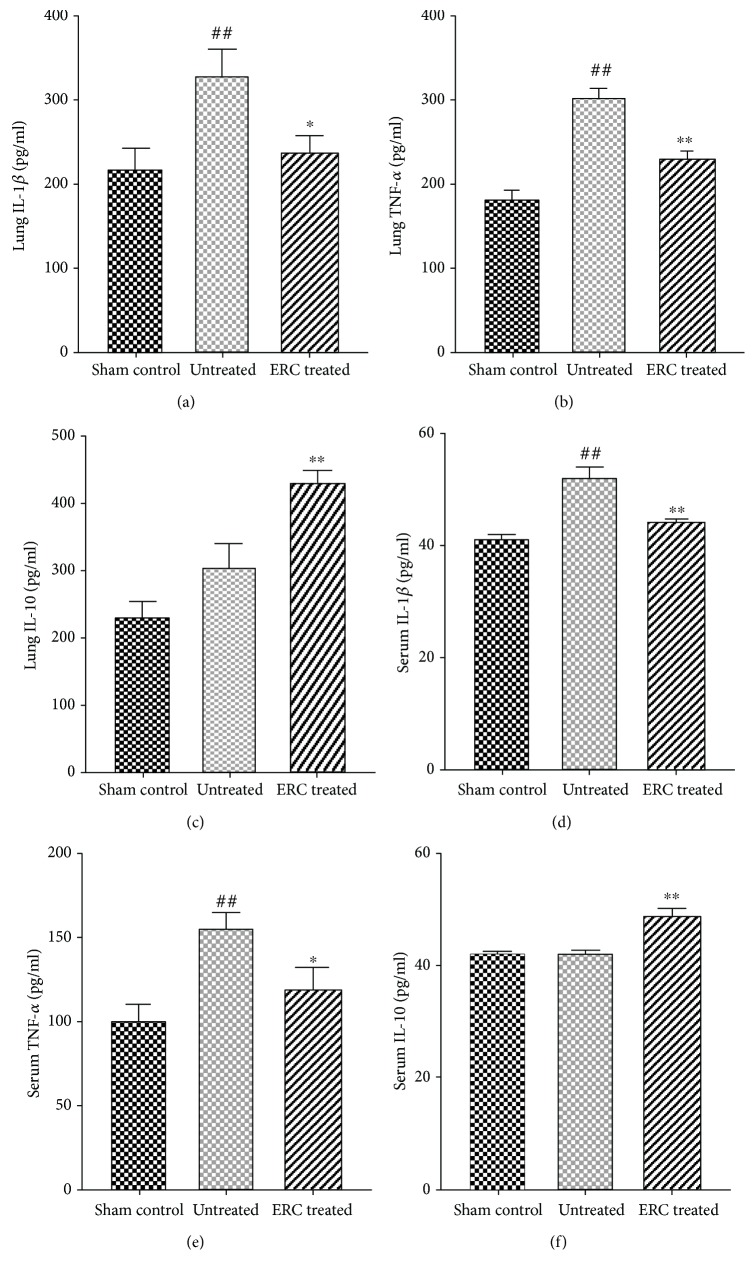
Treatment with ERCs diminishes pulmonary fibrosis by regulating cytokine profiles. The levels of IL-1*β*, TNF-*α*, and IL-10 in the lung and serum were detected by ELISA kit and compared among different groups. Data indicated that ERC treatment significantly decreased the levels of IL-1*β* and TNF-*α*, while it increased the level of IL-10 in both the lung and serum. Values represent mean ± SEM. (^##^*p* < 0.01 versus the sham control group. ^∗^*p* < 0.05 and ^∗∗^*p* < 0.01 versus the untreated group, *n* = 6). ERCs: endometrial regenerative cells; IL-1*β*: interleukin 1 beta; TNF-*α*: tumor necrosis factor-alpha; IL-10: interleukin 10.

To determine whether ERC treatment could affect cytokine profiles, the levels of local and serum inflammatory cytokines were analyzed and compared among different groups. For the levels of proinflammatory cytokines, we found that the levels of IL-1*β* and TNF-*α* in the ERC-treated group were much lower than that of the untreated group in both the lung (*p* < 0.05, Figures 6(a) and 6(b)) and the serum (*p* < 0.05, Figures 6(d) and 6(e)). Meanwhile, the level of anti-inflammatory cytokine IL-10 was elevated after ERC treatment as compared to the untreated group (*p* < 0.01, Figures 6(c) and 6(f)). These data indicated that treatment with ERCs may slow down the occurrence of fibrosis by suppressing the level of proinflammatory cytokines and enhancing the level of anti-inflammatory cytokines.

### 3.7. ERCs Attenuate Pulmonary Fibrosis by Upregulating Antiapoptotic and Antifibrotic Factors in the Lungs

**Figure 7:**
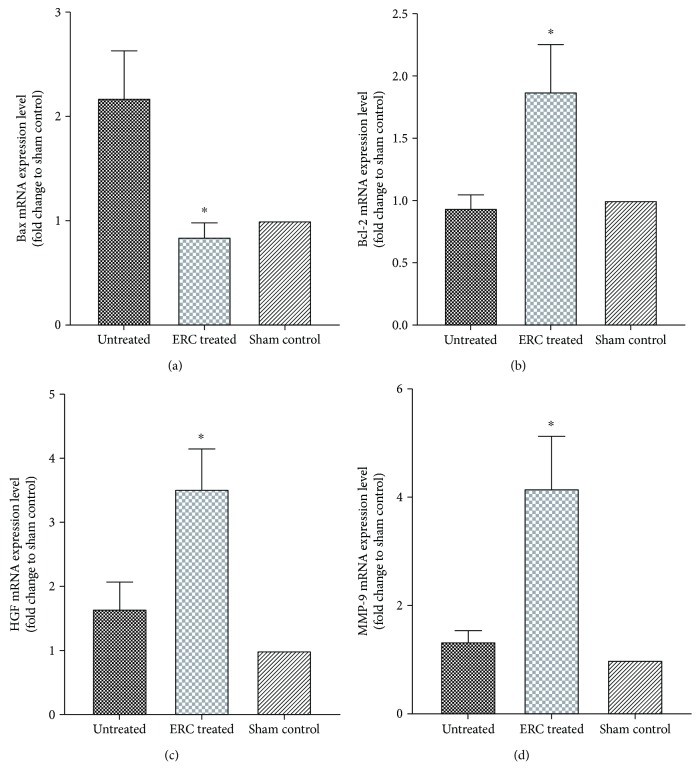
ERCs attenuate pulmonary fibrosis by upregulating antiapoptotic and antifibrotic factors in the lungs. RT-qPCR was performed to detect the relative expression levels of mRNAs of (a) Bax, (b) Bcl-2, (c) HGF, and (d) MMP-9. Data revealed that ERCs upregulate the expression of HGF, MMP-9, and Bcl-2, while downregulating the expression of Bax. Values were normalized to *β*-actin. Bar graphs represent mean ± SEM of three separate experiments. *p* values were determined by Independent-samples *t* test. Data shown are representative of three separate experiments performed. (^∗^*p* < 0.05 versus the untreated group, *n* = 6). ERCs: endometrial regenerative cells; RT-qPCR: quantitative real-time polymerase chain reaction; Bax: bcl-2-associated x protein; Bcl-2: b cell lymphoma-2; HGF: hepatocyte growth factor; MMP-9: matrix metalloproteinase 9.

To explore the underlying mechanisms of ERCs on alleviating fibrosis, we tested the mRNA levels of several possible targeting factors related to antiapoptosis and antifibrosis. As compared to the untreated group, we found that after treatment with ERCs, the expression of apoptotic factor Bax in the lung tissue was decreased (*p* < 0.05, Figure 7(a)), while the expression of antiapoptotic factor Bcl-2 was significantly increased (*p* < 0.05, Figure 7(b)). In addition, the antifibrosis factors HGF and MMP-9 were also markedly elevated after ERC treatment (*p* < 0.05, the untreated group versus the ERC-treated group, Figures 7(c) and 7(d)). The above results suggested that ERCs may play an important role in attenuating pulmonary fibrosis by upregulating antiapoptotic and antifibrotic factors.

## 4. Discussion

Idiopathic pulmonary fibrosis is a progressive disease whose incidence and mortality are increasing steadily worldwide [37, 38]. Currently, there is no effective treatment for this disease. MSCs have been used for scientific research and clinical treatment in search of new and diversified therapies, but invasiveness extraction and the proliferative capacity of MSCs have limited its development [22, 39, 40]. ERCs act as a new autologous source of stem cells and can be obtained in large amounts without karyotypic abnormalities [22]. Moreover, its high proliferation ability and easy access in obtaining provide an adequate source for treatment [24]. It has been reported that ERCs can differentiate into alveolar epithelial cells and secretion of MMP3 and MMP10 at 10–100,000-fold higher levels than mesenchymal stem cell lines [22]. Based on the above statement, we designed this study and discovered that ERC treatment is an effective therapeutic measure to alleviate pulmonary fibrosis. We focused on investigating the degree of fibroses by HE staining, Masson staining, and hydroxyproline quantification and then are mainly concerned with its anti-inflammatory, antiapoptotic, and antifibrotic effects.

ERCs could be located in the pulmonary circulation and play an important role. On account of a large number of blood flow through the lung and the attraction of pulmonary chemokine and inflammatory cytokines, once ERCs are injected into the vein, they would mostly be trapped and concentrated in the lung. Related studies have demonstrated that ERCs could be adsorbed to the lungs in in vivo and in vitro experiments [29].

The current disease paradigm is the ongoing AEC injury and apoptosis, deficiencies in regeneration of normal alveolar structure, aberrant lung repair, and fibroblast activation, thereby leading to progressive fibrosis. Taking into account the pathogenic role of bleomycin, we selected the second day after pulmonary fibrosis induction as the time point for ERC injection. The early ERC treatment could prevent a storm of inflammation; meanwhile, it could inhibit subsequent development of fibrosis and protect alveolar epithelial cells from injury. Thus, sequential responses of fibrosis caused by alveolar epithelial cell damage were prevented at the beginning of the disease [41, 42]. If ERCs are used for clinical treatment in the future, it may exhibit better outcome when ERCs are administered at the earlier time point after diagnosis. Although our data in this study revealed the efficacy of ERCs in alleviating lung fibrosis, the action mechanisms of ERCs in this case require further investigation. The follow-up studies will be designed to examine the antifibrosis and/or anti-inflammation of ERCs at multiple time points, so that we will understand if ERCs mainly inactivate the inflammation and/or suppress fibrogenesis.

At the same time, we speculated that ERCs could also play a crucial role by secretion of antifibrosis factors and transformation to alveolar epithelial cells while fibrosis occurs. Our experiment detected the changes of antifibrosis factors in the lung and confirmed that ERCs were indeed acting on the microenvironment of fibrosis. However, the in-depth mechanism study on whether ERCs transdifferentiated into alveolar epithelial cells and thereby resulted in alleviating fibrosis is warranted.

Mice received intratracheal injection of bleomycin in accordance with accepted methods [43]. During the modeling process, we used insulin needles to rapidly spray 50 *μ*l liquid and then immediately rotated the mice vertically to ensure that bleomycin was distributed into each lobes of the lung. However, bleomycin was injected only in one direction into the trachea, along with the influence of breath and gravity. We cannot guarantee that pulmonary fibrosis occurs uniformly in each lung lobe, but we insisted consistent operation in every modeling process. Each group was randomly assigned and the same lung lobe was taken for the same examination to eliminate the system error. Moreover, by contrast with the untreated group, we found that ERC treatment significantly attenuated inflammatory cell infiltration, alveolar structure destruction, and fibrous tissue formation in the lung tissue. By Masson staining, we could qualitatively and intuitively discover that the fibrotic tissue was markedly reduced in the ERC-treated group. By measurement of hydroxyproline, which accounted for around 13.5% of the fiber tissue content [44], we could precisely calculate that the ERC-treated group displayed a marked reduction of fibrous tissue.

Growing evidence indicated the role of oxidative stress in the pathophysiology of lung fibrosis [45, 46]. In this study, we found that bleomycin induced a marked increase of lipid peroxidation as indicated by the malondialdehyde (MDA) level and this level was significantly decreased upon ERC treatment. In addition, we found that total superoxide dismutase (T-SOD), playing an important role by scavenging superoxide anion radical and protecting cells from injury, was improved by the ERC injection, when compared to that of the untreated group. These findings suggest that ERCs may play a role in protecting lung tissue against oxidative stress injury.

The underlying mechanisms of IPF is complex, including stimulating secretion and recruitment of cytokines. It has been reported that MSCs have anti-inflammatory effects in liver, kidney, and lung fibrosis [47–49]. To investigate whether ERCs play an anti-inflammatory role in the development and progression of pulmonary fibrosis, we have measured the content of IL-1*β*, TNF-*α*, and IL-10 in the serum and the lung tissue. Among them, IL-1*β* is a proinflammatory regulatory factor involved in the regulation of inflammation and fibrosis, which is crucial in the early stage of pulmonary fibrosis [50, 51]. IL-1*β* has been previously shown to hamper silica-induced inflammation and fibrosis by inhibiting TNF-*α*, MCP-1, TGF-*β*1, collagen I, and fibronectin mediators and modulating the Th1/Th2 balance [52]. Besides, related researches have revealed that WNT/*β*-catenin signaling can induce IL-1*β* expression by alveolar epithelial cells in pulmonary fibrosis [53]. Then we could infer that, by downregulation of IL-1*β*, ERCs might provide a mild microenvironment for tissue repair. This agrees with the hypothesis that stem cells can ameliorate the microenvironment of the injury site [54, 55]. Cooperating with IL-1*β*, TNF-*α* also plays a synergistic role in promoting inflammatory cell adhesion, releasing inflammatory mediators, and enhancing lung injury.

Meanwhile, previous studies have shown that Ad-MSCs have autocrine cytokine IL-10 function, which can effectively prevent alveolar macrophages from releasing a variety of inflammatory and profibrosis factors, thereby preventing the cascade secretion of cytokines [56]. High levels of IL-10 also inhibit production and activation of TGF-*β*, thereby inhibiting the occurrence of inflammatory responses [57, 58]. In the present study, the level of IL-10 in the serum and the lung was elevated after ERC treatment, suggesting that ERCs might protect the mice from pulmonary fibrosis by upregulating IL-10 both locally and systematically.

Multiple lines of evidence have revealed that apoptosis and injury of alveolar epithelium play a primary role in pulmonary fibrosis, then recruiting cytokines, inflammatory cells, and fibrosis factors, thus leading to microenvironment disorder [41, 59, 60]. Bax, one of the most important apoptosis genes, encoded Bax protein which can form heterodimer with Bcl-2 or be homologous with itself, participating in the regulation of apoptosis. The present experiment revealed that the mRNA expression level of Bax/Bcl-2 in the ERC-treated group was significantly lower than that in the untreated group. Thus, we conclude that ERCs might protect alveolar epithelium cells from damage by antiapoptosis.

The vital role of TGF-*β* in promoting fibrosis has been proven in numerous studies [9, 61, 62]. Cells secreting TGF-*β* are much like alveolar macrophages, epithelial cells, and fibroblasts. Among them, alveolar macrophages and epithelial cells may be the major source of TGF-*β* [63, 64]. When tissue damage and infection occur, the microenvironment of the lung is broken and TGF-*β* begins to function as a central regulator [65]. Its main roles include (1) providing chemotactic effects on monocytes and macrophages and promoting the expression of cytokines related to pulmonary fibrosis such as TNF-*α*, IL-1, IL-6, and PDGF [66]; (2) inducing epithelial-mesenchymal transition in alveolar epithelial cells [61, 67]; (3) promoting the migration, proliferation, differentiation of fibroblasts, and deposition of extracellular matrix [68]; and (4) protecting the stability of the extracellular matrix without degradation. However, through the localization and qualitative analysis by immunohistochemistry in this study, the expression of TGF-*β* in the ERC-treated group was significantly lower than that in the untreated group. It reminds us that ERCs may play a role by potentially secreting cytokine or directly contacting macrophages and alveolar epithelial cells in the lung tissue and then inhibiting their secretion of TGF-*β*. Thus, lower expression of TGF-*β* mediated by ERC treatment helped in cutting off the source of fibrogenic factors, reducing pulmonary inflammatory cell aggregation and preventing the occurrence of EMT and thereby relieving pulmonary fibrosis.

We have also detected HGF and MMP-9 mRNA levels in lung tissue, and found that ERC treatment did increase the expression of these two antifibrosis factors. It is worth noting that HGF was once considered to be a hepatotropic factor; it is a newly discovered mesenchymal-derived multifunctional growth factor [69]. There is evidence that C-met, specific receptor of HGF, expresses in alveolar epithelial cells [70]. These results indicate that HGF is targeted to adjacent epithelial cells, regulating their proliferation and differentiation and thus promoting the structural remodeling of alveolar epithelium [70]. Moreover, matrix metalloproteinases (MMPs) are proteinases that can degrade all components of the extracellular matrix and numerous nonmatrix proteins [71, 72]. Among which, MMP-9 is one of the most significantly overexpressed proteins in the lungs of patients with IPF [73]. By detecting HGF and MMP-9, we have provided a potential target for ERCs to alleviate lung fibrosis.

In summary, these findings from our study indicate that human ERC-based therapy can attenuate bleomycin-induced pulmonary fibrosis in mice. Although the results are inspiring and encouraging, an in-depth study in investigating the signaling pathway and the interaction of microenvironment in ERC-mediated lung protection is warranted.

## 5. Conclusions

In this study, we for the first time have demonstrated that human ERCs, a new type of regenerative stem cells, have immunosuppressive and antifibrosis properties and play an important role in attenuation of bleomycin-induced pulmonary fibrosis in mice. We found that ERCs are effective in alleviating fibrosis, decreasing hydroxyproline content and fibrosis initiation factor TGF-*β* and upregulating antiapoptosis gene Bax and antifibrosis factors of HGF and MMP-1, while reducing the serum and lung homogenate of IL-1*β* and TNF-*α* and increasing those of IL-10. Taken together, the results generated in this study form a foundation for further study of ERC mechanism acting on pulmonary fibrosis and provide a rationale for the use of ERCs in clinical treatment.
